# The rapidly evolving monkeypox epidemic: A call to action to leave no one behind

**DOI:** 10.1371/journal.pmed.1004128

**Published:** 2022-10-31

**Authors:** Steffanie A. Strathdee, Davey M. Smith, Megan Halbrook, Placide Mbala-Kingebeni, Shira Abeles, Francesca Torriani, Anne Rimoin

**Affiliations:** 1 Division of Infectious Diseases and Global Public Health, Department of Medicine, University of California San Diego School of Medicine, San Diego, California, United States of America; 2 Fielding School of Public Health, University of California—Los Angeles, Los Angeles, California, United States of America; 3 Institut National de Recherche Biomédicale (INRB), Kinshasa, Democratic Republic of Congo; 4 University of Kinshasa, Kinshasa, Democratic Republic of Congo

## What are the known knowns?

**Fig 1:**
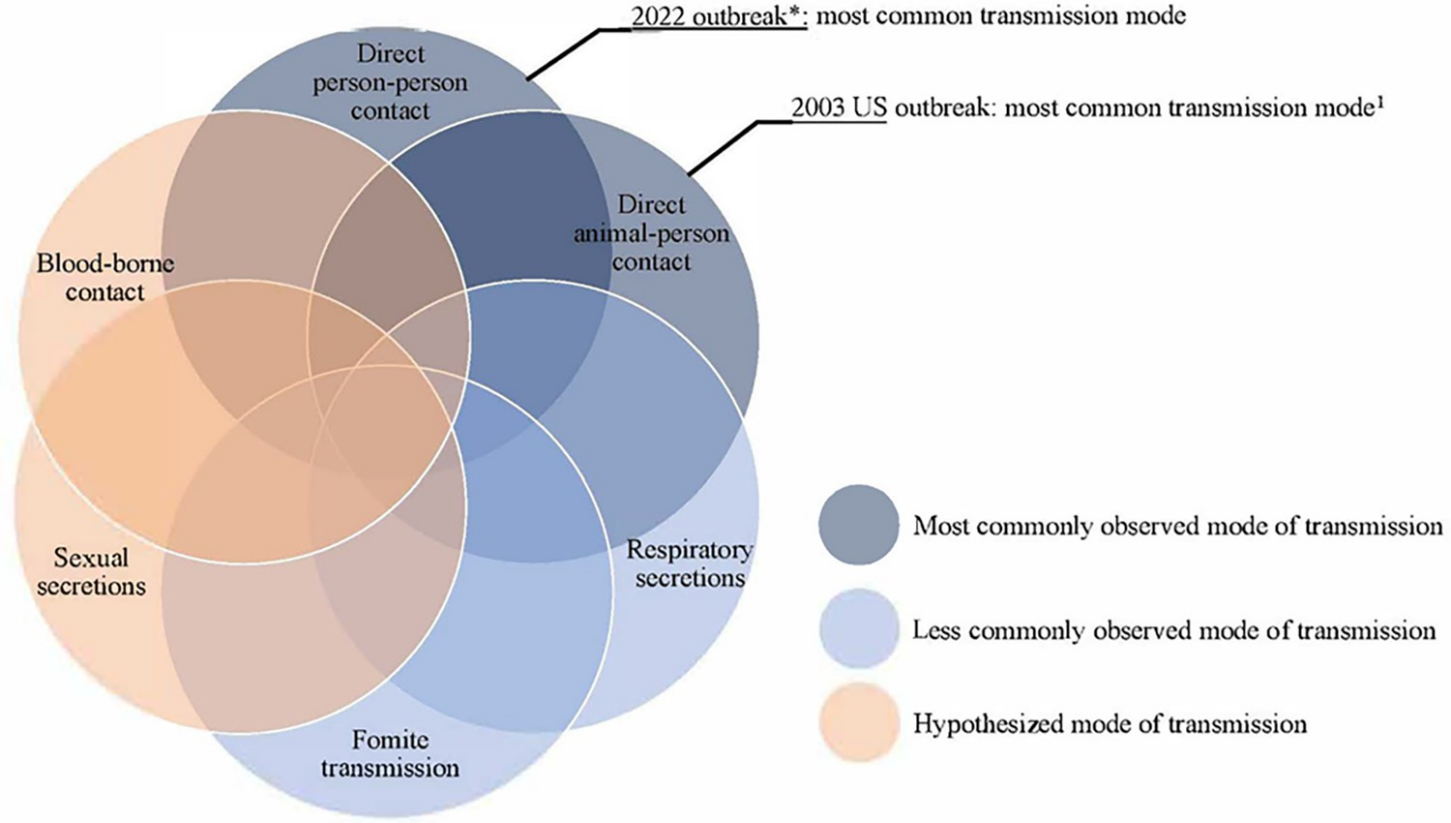
Known and putative risk factors for human monkeypox virus transmission. The MPXV that circulated in 2003 belongs to clade IIa, the current outbreak is associated with clade IIb [5].

Monkeypox virus (MPXV) is characterized by painful skin lesions and fever, sometimes with lymphadenopathy, pharyngitis and myalgia, and a prodromal period [1]. Although infections are self-limiting, severe complications can occur. In a multicountry case series, 13% were hospitalized, mostly for pain, but no one died [2]. Unlike previous MPXV outbreaks that were zoonotic and primarily confined to Africa [3], this pandemic had multiple introductions of a seemingly more transmissible Clade 3 B.1 variant in Western Europe and North America and is characterized by human-to-human transmission [4], mostly among men having sex with men (MSM) [2]. Although direct contact with infected lesions and fomites appear to be the most important risk factors (Fig 1), respiratory secretions can transmit MPXV and it can cross the placenta [5]. We can expect ongoing transmission since global population immunity waned following cessation of routine smallpox vaccination [3].

Vaccines exist. Single-dose and dual-dose smallpox vaccines seem efficacious against MPXV [6]. Both remain in short supply due to shortcomings in global manufacturing and distribution. Roll-out has been further complicated by lack of testing to inform decisions about which populations should be prioritized, which has contributed to vaccine inequities.

## What are the known unknowns?

We can predict spread. MPXV DNA has been detected in feces [7], nasopharyngeal swabs [8,9], urine [8,9], saliva [7,9], blood [8], and semen [2,7,9]. If it persists and is viable in sexual fluids, other at-risk populations include those engaging in sex work and sexual entertainment. If it persists in blood, it could be transmitted by contaminated paraphernalia of injection drug use. An outbreak has been associated with a piercing and tattoo parlor in Spain [10]. New animal reservoirs could include pets and other domesticated animals [3].

Spillover to other vulnerable populations should be anticipated due to close associations between MPXV and rodents, oral–fecal contamination, and transmission between close contacts. This includes people experiencing homelessness, incarceration, addiction, and forced migration due to intersecting risks such as poverty, stigma, discrimination, mobility, and low health literacy, which can foster medical mistrust and vaccine hesitancy. Minority groups who face systemic prejudices in healthcare settings are being disproportionately affected. Congregate settings such as homeless encampments, prisons/jails, nursing homes, daycares, and refugee/migrant camps are also at risk. Mathematical modeling scenarios can be used to predict trends in transmission, morbidity, and mortality to inform prevention efforts, as was done for HIV and Severe Acute Respiratory Syndrome Coronavirus 2 (SARS-CoV-2).

Potential treatment exists. Smallpox antivirals (tecovirimat and brincidofovir) may reduce disease severity and duration [11], but safety and efficacy data await results from clinical trials.

## We know enough to act

Learn from past successes. Smallpox was eradicated in 1980 following a globally coordinated plan involving testing, contact tracing, and ring vaccination. Similar approaches should be undertaken for MPXV, using sero-surveillance, molecular testing of high-risk populations, and wastewater surveillance.

Learn from past mistakes. Although MPXV is very different from SARS-CoV-2 and HIV, the Coronavirus Disease 2019 (COVID-19) and HIV/AIDS pandemics offer lessons. Testing, vaccination, and treatment should be immediately scaled up and offered free to high-risk populations globally, through coordinated strategies that minimize bureaucracy (e.g., strict eligibility requirements for testing) and includes timely contact tracing. Affordable health care, paid sick leave, and tele-health should be expanded. Health communication requires mandated reporting, consistent messaging and transparency, being clear about what we know and what we don’t. Concerted efforts should be made to quell stigma, misinformation, and disinformation through meaningful consultation with affected populations.

Address global health inequities. International multiagency coordination is needed so high-income countries do not hoard vaccines, treatments, or protect patents at the expense of lower and middle-income countries where MPXV is endemic. Global cooperation and political will is needed to assist lower income countries with access and last-mile delivery of testing, treatment, and vaccines. Affected populations need to be consulted at all stages to ensure that interventions are regionally and culturally appropriate.

Take action. Decriminalizing substance use, sex work, homosexuality, and homelessness would decrease stigma and encourage vulnerable populations to seek testing, improving case-finding, and treatment. During the COVID-19 pandemic, some regions depopulated prisons or included sex workers in government subsidies [12]. Regions facing armed conflict and other complex emergencies cannot be overlooked in prevention and treatment efforts. Testing and vaccination programs should leverage social media and engage nongovernmental organizations in mobile outreach.

Invest in research. Studies to characterize the epidemiology, social networks, clinical presentation, morbidity, and mortality associated with MPXV infection are critically needed to inform primary and secondary prevention. Sero-surveillance should extend to animals, including pets and wildlife that could serve as reservoirs [3]. Treatments found safe and effective should be evaluated as prophylaxis in high-risk populations. Research and implementation studies could leverage existing networks including but not limited to those established for HIV/AIDS (e.g., AIDS Clinical Treatment Group, Centers for AIDS Research, HIV Prevention Trials Network, HIV Vaccine Trials Network) and COVID-19 (e.g., ACTIV, RADx, RADxUP).

## What about the unknown unknowns?

MPXV isn’t the first pandemic of the 21st century, but it has already shown how unprepared we continue to be. Declaring a public health emergency of international concern (PHEIC) has limited impact if not met with action, building upon COVID-19 mitigation approaches, with an eye towards other threats (e.g., enteroviruses, avian influenza, new coronaviruses and antimicrobial resistance). Addressing root causes of syndemics that disproportionately affect socially disadvantaged populations could significantly reduce health disparities associated with multiple disease outcomes and would ultimately be cost saving. Ensuring that we leave no one behind requires global cooperation to strengthen infrastructures for public health surveillance and capacities of health care systems and their workers. Without addressing global health inequities, we will continue to be unprepared for future pandemics.

## References

[1] Breman JG, Kalisa R, Steniowski MV, Zanotto E, Gromyko AI, Arita I. Human monkeypox, 1970–79. Bull World Health Organ. 1980;58(2):165–182. 6249508 PMC2395797

[2] Thornhill JP, Barkati S, Walmsley S, Rockstroh J, Antinori A, Harrison LB, et al. Monkeypox Virus Infection in Humans across 16 Countries—April-June 2022. N Engl J Med. 2022;387(8):679–691. doi: 10.1056/NEJMoa2207323 35866746

[3] Simpson K, Heymann D, Brown CS, Edmunds WJ, Elsgaard J, Fine P, et al. Human monkeypox—After 40 years, an unintended consequence of smallpox eradication. Vaccine. 2020;38(33):5077–5081. doi: 10.1016/j.vaccine.2020.04.062 32417140 PMC9533855

[4] Reynolds MG, Davidson WB, Curns AT, Conover CS, Huhn G, Davis JP, et al. Spectrum of infection and risk factors for human monkeypox, United States, 2003. Emerg Infect Dis. 2007;13(9):1332–1339. doi: 10.3201/eid1309.070175 18252104 PMC2857287

[5] Jezek Z, Grab B, Szczeniowski MV, Paluku KM, Mutombo M. Human monkeypox: secondary attack rates. Bull World Health Organ. 1988;66(4):465–470. 2844429 PMC2491159

[6] Albarnaz JD, Torres AA, Smith GL. Modulating Vaccinia Virus Immunomodulators to Improve Immunological Memory. Viruses. 2018;10(3). doi: 10.3390/v10030101 29495547 PMC5869494

[7] Antinori A, Mazzotta V, Vita S, Carletti F, Tacconi D, Lapini LE, et al. Epidemiological, clinical and virological characteristics of four cases of monkeypox support transmission through sexual contact, Italy, May 2022. Euro Surveill. 2022;27(22). 10.2807/1560-7917.ES.2022.27.22.2200421 PMC9164671 35656836

[8] Adler H, Gould S, Hine P, Snell LB, Wong W, Houlihan CF, et al. Clinical features and management of human monkeypox: a retrospective observational study in the UK. Lancet Infect Dis. 2022;22(8):1153–1162. doi: 10.1016/S1473-3099(22)00228-6 35623380 PMC9300470

[9] Peiro-Mestres A, Fuertes I, Camprubi-Ferrer D, Marcos MA, Vilella A, Navarro M, et al. Frequent detection of monkeypox virus DNA in saliva, semen, and other clinical samples from 12 patients, Barcelona, Spain, May to June 2022. Euro Surveill. 2022;27(28). 10.2807/1560-7917.ES.2022.27.28.2200503 PMC9284919 35837964

[10] Del Rio GV, Palacios JG, Morcillo AM, Duran-Pla E, Rodriguez BS, Lorusso N. Monkeypox outbreak in a piercing and tattoo establishment in Spain. Lancet Infect Dis. 2022. 10.1016/S1473-3099(22)00652-1 PMC9534154 36183706

[11] Russo AT, Grosenbach DW, Chinsangaram J, Honeychurch KM, Long PG, Lovejoy C, et al. An overview of tecovirimat for smallpox treatment and expanded anti-orthopoxvirus applications. Expert Rev Anti Infect Ther. 2021;19(3):331–344. doi: 10.1080/14787210.2020.1819791 32882158 PMC9491074

[12] Strathdee SA, Abramovitz D, Harvey-Vera A, Vera CF, Rangel G, Artamonova I, et al. Prevalence and correlates of SARS-CoV-2 seropositivity among people who inject drugs in the San Diego-Tijuana border region. PLoS ONE. 2021;16(11):e0260286. doi: 10.1371/journal.pone.0260286 34807963 PMC8608290

